# Cases Illustrating the Effects of the Roller Bandage

**Published:** 1830

**Authors:** Joseph K. Sparks

**Affiliations:** Cincinnati


### Art. V.—*Cases illustrating the effects of the Roller-Bandage.*—By Joseph K. Sparks, M. D. of Cincinnati.

### Case I.

On the 24th of February, 1827, Amzi Leach came to my office, having a bad wound on his left hand. He said that fifteen days previous to that time, he fired off a gun that was overcharged, and the barrel bursted near the breech, which lacerated the palm of his left hand, and denuded the thumb-bone.

He was then in a boat on the Mississippi river. A physician was called to see him, who amputated the thumb and dressed the wound, which was not dressed again till I did it, fifteen days after. When he came to me the day above named, the wound extended from the flexor tendons of the ring finger, half round the first joint of the middle finger, and to the first joint of the fore finger, from thence over the stump of the thumb and round on the back of the hand to the extensor tendon of the middle finger. The muscle of the middle finger had sloughed off from the first to the second joint, on the side that looks towards the ring finger. The middle finger was swoln and œdematose, the back part and the side which looks towards the fore finger, being the only parts through which the blood could circulate to the end of the finger. The fore finger was likewise swollen, but no part had sloughed off, except at the first joint, towards the palm of the hand. This wound had been without dressing so long, and had suppurated so much, that it was offensive to the people as they passed him in the streets.

I washed it with soap suds and spread dry lint over the whole suppurating surface, and applied the roller-bandage, with a view to increase the circulation of blood in the œdematose parts.

The next day, 25th February, 1827, when I took the dressing off, the middle finger had a more healthy appearance, and the blood seemed to circulate in every part where there was any muscle, and the rest of the wound looked proportionably well. I cleansed it with the soap suds and applied the bandage in the same way.

26th. Dressed it and applied the lint and bandage.

27th. I called in three neighboring physicians—they all said that it would be better to amputate the fore and middle fingers, but it might be deferred longer, as the inflammation had subsided. They likewise recommended the yeast poultice to be applied to the wound—as I had none at hand I dressed it with the lint and bandage again.

28th. The wound looked very well, and I presumed that it could not improve faster; as the suppuration was perfectly healthy and granulations had commenced very beautifully. I therefore continued the same application.

March 1st. I saw that the flexor sublimus perforatus, extending to the middle finger was dead, which in a few days sloughed off.

I continued the lint and the bandage till the 10th of March, at which time the wound was nearly filled with granulations and covered for the most part with new skin. At this time he did me the favor to abscond without giving me further trouble, and I do not know the final termination of the case.

Having seen a man's hand lacerated by the bursting of a gun barrel when I was a student of medicine, and having witnessed the treatment, which was the application of poultices, and several strata of cloths round the part, I am prepared to say, without being prejudiced against the former treatment, or enthusiastically in favor of the latter; that the bandage and the dry lint were the best applications, in two respects:—

First, the bandage supported the swollen parts so as to promote the circulation.

Secondly, it did not produce any unnecessary heat or suppuration.

### Case II.

Jacob Varley, about twenty years old, came to my office, 30th December, 1828, with his hand very much inflamed and exceedingly painful. Some four or five days previous he had received a slight hurt on the end of his little finger while at work in a pork house. The weather being very cold, it inflamed immediately.

When he came to me the entire hand was very much swelled, and the pain acute. I bathed it in warm water half an hour or more, gently pressing it with my hand, till the part was considerably softened; I then applied the roller bandage, beginning at the end of the fingers, and continuing it to the shoulder.

I left him about an hour after dark. Next morning, 31st December, the little finger from just below the second joint to the end, was gangreous. I then put on a blistering plaster extending over the palm and back of the hand, and applied the bandage over it, from the ends of the fingers to the shoulders as I had done before.

January 1, 1829. The little finger had mortified as far as it was gangrenous the day before. I dressed the part that was blistered and applied the bandage with a corn meal poultice over it.

2nd The gangrene appeared to be arrested, except a small place in the palm of the hand about the size of a pea, three fourths of an inch from the first joint of the little finger. I then dissected the little finger at the first joint which was a little below the part that was mortified. As the inflammation subsided, I dressed the hand like other blistered surfaces, and kept the integuments in contact over the dissected part. I continued the dressing daily, till 10th January, at which time it was so well as to require my attention no longer.

His hand got well and he worked on a farm through the spring; I have seen him twice since, and it is perfectly sound.

Before I saw J. V. he had poulticed his hand, but was not benefited at all by the application. I am certain that much good was done in the first instance by bathing it in warm water, and gently pressing the swelled part till it softened, when the pain abated considerably. But I feel safe in saying, that, had it not been for the use of the bandage, that gangrene and mortification would have extended into the hand.

January 15, 1830.

